#  Aplicação do saber científico: a translação do conhecimento em um instituto de ciência e tecnologia em saúde pública

**DOI:** 10.1590/0102-311XPT006523

**Published:** 2023-12-04

**Authors:** Ângela Maria Andrade Scavuzzi, Valdeyer Galvão dos Reis, Marcelo Santos Ramos, Maria Julia Alves de Souza, Ingrid Winkler, Camila de Sousa Pereira-Guizzo

**Affiliations:** 1 Instituto Gonçalo Moniz, Fundação Oswaldo Cruz, Salvador, Brasil.; 2 Centro Universitário SENAI CIMATEC, Salvador, Brasil.

**Keywords:** Translação de Conhecimento, Gestão de Ciências, Tecnologia e Inovação em Saúde, Ciência da Implementação, Knowledge Translation, Health Sciences, Technology, and Innovation Management, Implementation Science, Traslación del Conocimiento, Gestión de Ciencia, Tecnología e Innovación en Salud, Ciencia de la Implementación

## Abstract

A translação do conhecimento (TC) tem como propósito a utilização prática dos resultados de pesquisas científicas e o monitoramento dos benefícios causados à saúde da população. Na área de saúde, o governo e, principalmente, a sociedade esperam que os investimentos em pesquisas obtenham resultados que vão além da produção e da publicação do conhecimento, e provoquem soluções como políticas públicas, sistemas, produtos e tecnologias para beneficiar a saúde da população. Contudo, verifica-se ainda a necessidade de superar diversos desafios para eliminar as lacunas existentes entre a investigação e a aplicação. O objetivo deste estudo é propor estratégias, com base na identificação de barreiras e fatores facilitadores de um instituto de ciência e tecnologia (ICT) em saúde, para fomentar o processo de transformação do conhecimento científico, gerado nas pesquisas, em ações e produtos que contribuam para a melhoria da saúde da população. Os relatos das entrevistas, realizadas com 16 pesquisadores, permitiram a identificação de 10 categorias de barreiras, tendo destaque: “financiamento em ciência, tecnologia e informação (CT&I) limitado” e “apoio técnico insuficiente para a translação do conhecimento”. “Infraestrutura e apoio institucional” foi a categoria de fatores facilitadores mais citada pelos participantes. Por fim, foi desenvolvido o artefato “estratégias e abordagens para superação de barreiras à implementação de resultados de pesquisa”. Entre as estratégias, sugere-se a inclusão de uma disciplina de TC nos programas de pós-graduação *stricto sensu* e a criação de uma instância na estrutura organizacional do ICT voltada à prestação de suporte técnico e gerencial à aplicação de resultados de pesquisa.

## Abstract (en)

Knowledge translation (KT) aims at the practical use of scientific research results and at the monitoring of the benefits caused to the population’s health. In health, the government and especially society expect that investments in research will produce results that go beyond the production and publication of knowledge, provoking outcomes such as public policies, systems, products, and technologies to benefit the health of the population. However, closing the gaps between research and application requires overcoming a number of challenges. This study aimed to propose strategies to foster the process of transforming the scientific knowledge generated in research into actions and products that contribute to improving the population’s health based on the identification of barriers and facilitating factors of a health science and technology institute. The reports of interviews conducted with 16 researchers showed 10 categories of barriers, especially: “limited funding to the science and technology institute” and “insufficient technical support for knowledge translation”. “Infrastructure and institutional support” was the facilitating factor category participants mentioned the most. Finally, we developed the artifact “strategies and approaches for overcoming barriers to implement research results”. Among the strategies, we suggest the inclusion of a knowledge translation discipline in stricto sensu graduate programs and the creation of an instance in the organizational structure of the science and technology institute to technically and managerially support the application of research results.

## Resumen

La traslación del conocimiento (TC) tiene como propósito el uso práctico de los resultados de investigaciones científicas y el seguimiento de los beneficios causados a la salud de la población. En el área de la salud, el gobierno y, sobre todo, la sociedad esperan que las inversiones en investigaciones obtengan resultados que vayan más allá de la producción y publicación de conocimiento, y provoquen resultados, como políticas públicas, sistemas, productos y tecnologías en beneficio de la salud de la población. Sin embargo, se observa aun la necesidad de superar diversos desafíos para eliminar las brechas entre la investigación y la aplicación. El objetivo de este estudio es proponer estrategias con base en la identificación de barreras y factores facilitadores de un instituto de ciencia y tecnología (ICT) en salud, para fomentar el proceso de transformación del conocimiento científico generado en las investigaciones en acciones y productos que contribuyan a mejorar la salud de la población. Los relatos de las entrevistas a 16 investigadores permitieron identificar 10 categorías de barreras, con énfasis en: “financiación en CT&I limitado” y “apoyo técnico insuficiente para la traslación del conocimiento”. “Infraestructura y apoyo institucional” fue la categoría de factores facilitadores más citada por los participantes. Finalmente, se desarrolló el artefacto “estrategias y enfoques para la superación de barreras a la implementación de resultados de investigación”. Entre las estrategias, se sugiere la inclusión de una asignatura de TC en los programas de posgrado *stricto sensu* y la creación de una instancia en la estructura organizacional del ICT orientada a brindar apoyo técnico y gerencial a la aplicación de los resultados de la investigación.

## Introdução

O Brasil foi responsável por uma crescente produção de artigos científicos ao longo do período de 2000 a 2020, atingindo, no ano de 2020, a quantidade de 89.241 publicações, o que representa 2,76% da produção mundial. Esse número leva o país a ocupar a 13ª posição na produção de publicações científicas, sendo a área de Ciências da Saúde seu principal campo de estudo, representando 33% das publicações ^1^. Considerando a relação publicação-patentes como um dos indicadores de capacidade de inovação, o depósito e concessão de patentes demonstram que a competência de utilizar o conhecimento produzido leva o país a ocupar a 64ª colocação no ranking de países inovadores, de acordo com o índice global de inovação (GII) de 2018, entre 126 países avaliados; e a 57ª posição entre os 132 países incluídos no GII de 2021 ^2^^,^^3^. Os dados apresentados corroboram a literatura sobre o tema ao afirmar que criar, gerir e compartilhar tais conhecimentos por meio de publicações em periódicos ou apresentações em eventos científicos, apesar de necessário, não é suficiente para melhorar a prestação de serviços na área da saúde ou para efetuar um processo de tomada de decisão mais adequado ^4^^,^^5^^,^^6^.

Em 2008, Montagner ^7^, citando Bourdieu, afirmou que as pesquisas devem seguir a lógica do mundo acadêmico e científico, porém as necessidades da sociedade devem ser consideradas. Nessa lógica, a pesquisa científica deve ser planejada para ser utilizada e aplicada. Neste estudo, a utilização dos resultados de pesquisa é assumida como a aplicação do conhecimento produzido ou, ainda, a utilização de evidências científicas para superar a lacuna entre a criação e a transformação do conhecimento em ações que provoquem benefícios à saúde da população. De acordo com os autores Lavis & Mattison ^8^, as investigações devem ser focadas não apenas na descoberta, mas também na ação e na inovação. Para a Organização Mundial da Saúde (OMS), a inovação em saúde consiste em desenvolver e fornecer políticas, sistemas, produtos, tecnologias, serviços e métodos de saúde, novos ou melhores, para aprimorar a saúde das pessoas. Além disso, tais inovações podem ser consideradas em cuidados preventivos, promocionais, terapêuticos, de reabilitação e/ou assistivos ^9^.

Nos países desenvolvidos, as organizações de saúde estão sendo incentivadas a implementar práticas inovadoras baseadas em evidências científicas ^10^. A evidência científica é definida por Rycroft‐Malone & Stetler ^11^ como o conhecimento que é produzido sistematicamente, isto é, obtido de tal forma que é replicável, observável, acreditável, verificável ou basicamente sustentável. Na visão dos autores Lavis et al. ^12^, as evidências científicas são fontes de novos conhecimentos gerados e que devem ser utilizadas para apoiar as ações e procedimentos relacionados à saúde.

Buscando superar as dificuldades da trajetória entre o conhecimento produzido nas pesquisas e sua aplicação, surgem as teorias da translação do conhecimento, termo originado no Canadá como *knowledge translation*, adotadas pela OMS e por outros países e instituições ^13^. Ainda que essa abordagem possa estar associada a termos como “tradução do conhecimento”, “gestão do conhecimento”, “gestão do desempenho”, “incorporação de tecnologias”, “construção de capacidade organizacional”, entre outros, optou-se, neste estudo, por translação do conhecimento (TC).

Um número significativo de barreiras a serem ultrapassadas nesse processo é descrito por diversos autores encontrados na literatura sobre o tema. Dobbins et al. ^14^ destacam os seguintes limitadores da TC: falta de acesso a evidências de pesquisa atualizadas; habilidades limitadas de avaliação crítica por parte daqueles que decidem; excessiva quantidade de revisões; ambiente de trabalho que não facilita a transferência e a apropriação dos resultados de pesquisa; falta de autoridade na tomada de decisões para implementar resultados de pesquisa; resistência a mudanças; e recursos limitados de implementação.

Haines et al. ^15^ identificaram as barreiras potenciais à TC em sete diferentes ambientes: sistema de saúde (falta de recursos financeiros, incentivos financeiros inapropriados); recursos humanos (inadequações referentes à quantidade e qualidade); ambiente de prática (limitações de tempo, falta de organização dos registros); ambiente educacional (currículo escolar falho, educação continuada inapropriada ou inexistente, falta de incentivo para participar de atividades educacionais); ambiente social (influência da mídia criando demandas ou crenças inapropriadas, modismos e tendências, desvantagens de acesso e de competência informacional e comportamentos de saúde); ambiente político (ideologia inconsistente com evidências científicas, corrupção, pensamento de curto prazo); profissional médico (conhecimento obsoleto, influência da opinião de especialistas importantes, crenças e atitudes); pacientes (demanda de cuidados ineficazes, percepções e crenças culturais sobre o cuidado).

Nutley et al. ^16^ afirmam que, além das evidências não serem acessíveis para os tomadores de decisão, eles não têm tempo disponível para realizar buscas, avaliações e aplicação dos resultados de pesquisa. Dias et al. ^5^ referem-se à escassa comunicação e colaboração entre os pesquisadores e os que tomam decisões como uma dificuldade que precisa ser ultrapassada. Pearson et al. ^17^ destacam como dificuldades a distância entre: a pesquisa teórica, epidemiológica e de laboratório e a pesquisa clínica; a aplicação clínica dos resultados da pesquisa e a adoção de condutas, ações e políticas de saúde; a necessidade de conhecimento dos pacientes, dos profissionais de saúde, dos governos e das instituições e o trabalho que fazem os pesquisadores.

Em estudo realizado por Mahendradhata & Kalbarczyk ^18^, foi verificado que, especialmente nos países de baixa e média renda, as instituições acadêmicas estão enfrentando grandes desafios para realizar o processo de TC no enfrentamento da pandemia provocada pela COVID-19. Os autores afirmam que, embora tenham sido apresentados, em alguns estudos, as barreiras e os fatores facilitadores para a utilização prática do conhecimento em ambientes de poucos recursos, pouco foi discutido sobre as atividades de TC conduzidas por instituições acadêmicas durante uma emergência de saúde global complexa e sobre como essas instituições podem estar mais preparadas no futuro para conduzir o processo de utilização do conhecimento em outras situações, sejam emergenciais ou não. Para os autores, além da falta de conhecimento sobre o que é TC e como fazê-la, a limitação de recursos, a insuficiência de apoio institucional e a falta de adesão de líderes fazem com que esses países enfrentem barreiras adicionais, como a necessidade de habilidades sociais e os desafios no desenvolvimento de redes robustas, tanto internas quanto externas. Sendo assim, os autores concluem que a pandemia de COVID-19 aumentou a demanda por conhecimento para apoiar a tomada de decisão em vários aspectos e revelou o baixo nível de prontidão das instituições acadêmicas para realizar atividades de TC.

Oelke et al. ^19^ afirmam que existem muitos desafios para se implementar a TC no Brasil, incluindo a falta de familiaridade com o tema, dificuldades em identificar problemas de pesquisa relevantes, pouco envolvimento dos principais interessados, falta de parceria entre pesquisadores e usuários do conhecimento no processo de investigação, baixos orçamentos para pesquisa e pouco enfoque em TC pelas agências de financiamento. Os autores afirmam, também, que são necessárias pesquisas futuras para adaptar modelos teóricos de TC para o contexto brasileiro e para estudar as suas abordagens inovadoras, a fim de fomentar a utilização de resultados de pesquisa. Além desses desafios, os autores defendem que é preciso maior alinhamento entre as necessidades do sistema de saúde e as pesquisas que geram conhecimento científico, bem como maior direcionamento dos investimentos para problemas relevantes à saúde. Dessa forma, os autores concluem que, no Brasil, a TC é um ponto crítico devido à pouca disponibilidade de informação sobre o tema.

Somada aos desafios já apresentados, a fragmentação do conhecimento produzido em uma quantidade de pesquisas cada vez mais crescente, o custo de decisões inadequadas e a lentidão no processo de transformação do conhecimento em prática tornam a TC um campo primordial para a saúde pública ^20^. Ainda no contexto nacional, existem questões complexas e enormes iniquidades que causam a perda de oportunidades de colocar o conhecimento em prática e geram consequências indesejáveis na assistência à saúde ^21^.

A identificação dos fatores que impactaram e que continuam impactando, de forma positiva ou negativa, a TC gerada nas pesquisas realizadas em um instituto de ciência e tecnologia (ICT) em saúde, pertencente à estrutura organizacional de uma fundação ligada ao Ministério da Saúde, contribuirá para responder à questão de partida deste estudo: como ampliar a capacidade do ICT de transformar os conhecimentos gerados em práticas inovadoras que possam beneficiar a saúde da população brasileira. Andrade & Pereira ^22^ apontam que um maior investimento na capacitação de pesquisadores brasileiros em implementação do conhecimento é considerado relevante para a melhoria desse campo. Essa preocupação é reforçada quando analisamos que cada instituição apresenta um contexto distinto que afeta de forma positiva ou negativa o processo e os resultados da TC ^23^. Apesar do crescente interesse em estudos sobre como transformar o conhecimento em prática, poucas ideias e recomendações foram disponibilizadas na última década ^24^.

Diante do exposto, o objetivo geral deste estudo foi, portanto, propor estratégias, com base na identificação de barreiras e facilitadores de um ICT em saúde, para fomentar o processo de transformação do conhecimento científico, gerado nas pesquisas, em ações e produtos que contribuam para a melhoria da saúde da população.

## Método

Neste estudo, foi utilizada a abordagem *Design Science Research* (DSR), que tem como finalidade a construção de artefatos inovadores que se traduzam em benefícios para as organizações ^25^. O conceito de artefato utilizado nessa abordagem vai além de objetos físicos, uma vez que um artefato pode ser algo projetado, um engenho, uma artificialidade etc. Em princípio, qualquer coisa projetada para alcançar um objetivo pode ser considerada um artefato ^26^. A DSR é uma metodologia para se alcançar uma solução para diversos problemas de pesquisa. Essa forma de produção científica encontra-se situada entre as abordagens tradicionais, de caráter prescritivo, e o conhecimento prático para a solução de problemas em contextos reais ^27^, adequando-se ao propósito deste estudo.

A abordagem DSR tem diferentes etapas, iniciando com a identificação e conscientização do problema; proposição de artefatos para resolver o problema específico; projeto e desenvolvimento do artefato; avaliação; e contemplando, inclusive, a comunicação dos resultados ^27^^,^^28^^,^^29^^,^^30^^,^^31^^,^^32^^,^^33^^,^^34^^,^^35^^,^^36^^,^^37^^,^^38^. Neste estudo, apresentam-se os resultados relacionados às fases iniciais de identificação e conscientização do problema, bem como a proposição de artefatos, que foram denominadas, aqui, de estratégias.

Esta investigação enquadra-se como uma pesquisa qualitativa do tipo estudo de caso, que responde às perguntas sobre “como” e “por que”, a partir de um problema de pesquisa ^28^. Coerente com as características dos métodos qualitativos de pesquisa, buscou-se a compreensão do significado da TC para uma amostra específica de pesquisadores experientes a fim de entender profundamente as percepções desse grupo e os aspectos centrais para o desenvolvimento dos artefatos em um determinado contexto, permitindo, ainda, que o conhecimento produzido possa ser testado com outros grupos e em novos casos em estudos futuros ^36^. Embora não haja forte tradição de métodos qualitativos e da DSR em pesquisas na área da saúde, nota-se aumento no interesse por essas abordagens em investigações que buscam recomendações daqueles que vivenciam o objeto de estudo para introduzir inovações em suas organizações ou atividades ^25^^,^^36^^,^^37^^,^^38^.

### Definição da população e caracterização da amostra

Em um universo de 47 servidores em atividade de pesquisa no ICT, 25 integraram a população deste estudo, pois atenderam a pelo menos um dos dois critérios de inclusão descritos a seguir: critério 1 - pesquisadores que recebem bolsa de produtividade do Conselho Nacional de Desenvolvimento Científico e Tecnológico (CNPq); e critério 2 - pesquisadores que estão desempenhando a função de líder de laboratório de pesquisa.

Dessa população, 15 pesquisadores participaram do estudo e assinaram o Termo de Consentimento Livre e Esclarecido (projeto aprovado pelo Comitê de Ética em Pesquisa do SENAI CIMATEC - parecer nº 5.096.148). Esses indivíduos têm, majoritariamente, formação em Medicina, correspondendo a 47% da população. Os pesquisadores com formação em Ciências Biológicas (20%) e Farmácia (20%) também se destacam na representatividade e, junto aos formados em Medicina, compreendem 87% dos servidores em atividade de pesquisa aptos a participarem deste estudo. Destaca-se que a participação na pesquisa foi voluntária e confidencial, respeitando todas as diretrizes e normativas vigentes sobre a realização de pesquisas com seres humanos no Brasil.

### Coleta e análise de dados

O roteiro de entrevista foi inspirado no modelo proposto na estrutura consolidada para pesquisa de implementação, desenvolvida por Damschroder et al. ^29^. Foram considerados e adaptados três dos cinco domínios propostos pelos autores: características dos resultados de pesquisa (Domínio I - DI), cenário interno (Domínio II - DII) e cenário externo (Domínio III - DIII). Foram elaboradas 15 questões distribuídas entre esses três domínios, buscando-se identificar, na percepção dos entrevistados, quais fatores vêm atuando, positiva ou negativamente, na implementação dos resultados obtidos nas pesquisas realizadas no ICT.

Neste estudo, o DI representa a busca por informações relativas às especificidades de cada pesquisa finalizada que teve seus resultados efetivamente aplicados em benefício da população, bem como daquelas que, apesar de terem gerado conhecimento com potencial de causar impacto positivo imediato na saúde da população, deixaram de ser implementadas (exemplo de questão: Houve tentativa de implementação dos resultados obtidos nesta pesquisa? Descreva as dificuldades que impediram a implementação). O DII concentra-se no cenário interno do ICT estudado (exemplo de questão: Indique quais são as principais dificuldades à implementação dos resultados obtidos nas pesquisas relacionadas ao ambiente interno do ICT tais como infraestrutura, cultura, apoio etc.); enquanto o cenário externo do ICT é tratado no DIII (exemplo de questão: Indique as principais dificuldades encontradas no ambiente externo para a implementação dos resultados obtidos nas pesquisas, tais como, agências de fomento, ambiente político, órgãos reguladores etc.). É importante comentar que houve um estudo piloto do roteiro de entrevista, antes da coleta de dados para esta pesquisa, buscando verificar a clareza das perguntas e encadeamento do roteiro, propiciando a experiência da primeira autora nessa técnica de coleta de dados.

Utilizando as técnicas recomendadas no modelo de análise de conteúdo de Bardin ^30^, a gravação do conteúdo das entrevistas passou por um minucioso processo de categorização. Inicialmente, os relatos foram analisados e agrupados de acordo com a semelhança da ideia central das respostas. Posteriormente, cada agrupamento recebeu uma denominação, permitindo a categorização e a definição de cada uma delas. Após esse processo, foi possível identificar em qual dos três domínios - apresentados por Damschroder et al. ^29^ - cada categoria se enquadra. Na fase final da análise de conteúdo, as categorias identificadas foram refinadas considerando a ponderação entre os pesquisadores. As categorias, como ficaram definidas na versão final, representam um consenso entre os pesquisadores deste estudo e dois avaliadores externos, especialistas na área de gestão em saúde com mais de 10 anos de experiência no assunto.

O processo de categorização dos determinantes contextuais, que foram citados pelos pesquisadores como fatores que influenciam, positiva ou negativamente, a implementação dos resultados de pesquisas, exigiu um esforço de abstração a partir de fatos da realidade estudada que são únicos em si. Isto é, muitos fatos específicos e individuais foram agrupados e transformados em um número reduzido de conceitos. Foram definidas categorias referentes aos desafios (barreiras) que precisam ser ultrapassados na implementação de resultados de pesquisa; fatores facilitadores utilizados pelos servidores em atividade de pesquisa entrevistados nesse processo e que poderão ser potencializados; estratégias encontradas e sugeridas por esse público para serem utilizadas em maior escala no ICT. Assim, a categorização dos determinantes contextuais (barreiras e facilitadores) foi uma etapa fundamental na construção das estratégias propostas ao final deste estudo.

## Resultados e discussão

Os determinantes contextuais identificados e apresentados nas Tabelas 1 e 2 podem influenciar a implementação de resultados de pesquisa no contexto estudado, permitindo a construção e adequação de estratégias e abordagens (Quadro 1). Essas estratégias buscam diminuir as lacunas entre a pesquisa e a ação, possibilitando a ultrapassagem das barreiras e o fortalecimento dos fatores facilitadores ^23^. A Tabela 1, referente às dificuldades mencionadas pelos pesquisadores entrevistados, apresenta 10 categorias de barreiras à TC, agrupando a ideia central extraída dos relatos das entrevistas. Ainda na Tabela 1, é possível visualizar a definição de cada categoria, um exemplo de relato e a frequência de relatos em cada categorização e sua distribuição por domínio. No total, 291 relatos referentes às barreiras foram mencionados pelos participantes.

**Tabela 1:** Categorias de barreiras à translação do conhecimento (TC) por domínio.

Categorização	Definição	Exemplos de relatos	DI	DII	DIII	Total	%
Financiamento em CT&I limitado	Refere-se à insuficiência de recursos financeiros para a pesquisa, desenvolvimento e inovação	“*Existe a necessidade de um novo financiamento para implementar resultado da pesquisa*”	10	19	15	44	15,1
Apoio técnico insuficiente para TC	Refere-se à necessidade de recursos humanos com conhecimentos técnicos especializados para o suporte à TC no ICT	“*Falta apoio de um grupo que falasse olha identificamos uma série de potenciais produtos ou potenciais ideias para serem translacionadas e vender*”	8	21	8	37	12,7
Cooperação e parcerias restritas	Refere-se à necessidade de ampliar colaborações estratégicas, formais e informais, intra e extramuros, em apoio à implementação dos resultados de pesquisas	“*Não se entende que essas parcerias públicas-privadas são importantes. Existe um olhar torto para o pesquisador que busca a iniciativa privada para desenvolver seus projetos*”	6	17	12	35	12,0
Falta de formação e desenvolvimento de competências para TC no ICT	Refere-se à necessidade de aprimorar o processo de recrutamento, desenvolvimento e retenção de recursos humanos para atuar na implementação dos resultados das pesquisas	“*Nós pesquisadores não temos formação para inovação assim é muito difícil hoje ser pesquisador no Brasil*”	12	21	2	35	12,0
Necessidade de gestão das relações institucionais e governamentais com foco em TC	Refere-se à necessidade de fortalecer as interlocuções institucionais com entes públicos e privados, objetivando facilitar a implementação dos resultados das evidências científicas produzidas pelo ICT	“*Você desenvolve um teste e aí para esse teste chegar lá no leito do paciente não depende mais do pesquisador. É essa estrutura que precisa ter*”	7	2	18	27	9,3
Falta de critérios técnico-científicos na definição quanto à vocação de espaços físicos	Refere-se à necessidade de definição quanto à vocação e utilização de equipamentos e espaços físicos, segundo critérios técnicos que assegurem maior qualidade aos experimentos científicos	“*Não há uma sala de cultura adequada para trabalhar. Não existe a divisão por vírus, bactérias, parasitas etc. salas de cultura segmentadas*”	3	21	2	26	8,9
Baixa competência institucional para lidar com órgãos reguladores	Refere-se à competência institucional limitada para lidar com a complexidade das exigências normativas e legais por parte dos órgãos reguladores, o que facilitaria a TC	“*Precisa de muito mais aporte de recurso, precisa de gente que entenda de regulatório, falta de equipe treinada*”	10	9	5	24	8,2
Conflito entre os macroprocessos de pesquisa e gestão	É a incompatibilidade do modelo de gestão pública nacional com as especificidades das instituições de pesquisa científica, além de uma cultura desalinhada entre a gestão e a pesquisa que nem sempre consegue atender, com agilidade e eficiência, as demandas estratégicas das atividades meio e fim	“*Nós temos muitas restrições dentro do ambiente público. Não foi em vão que foram criadas fundações*”	3	14	6	23	7,9
Cultura de criatividade e inovação insuficientes	Refere-se à necessidade de incentivar a cultura de criatividade e inovação entre gestores e pesquisadores do ICT, bem como fortalecer a aproximação com o governo, a indústria, a sociedade e outras partes interessadas para promover a geração de ideias, conhecimentos, produtos e serviços, ampliando a capacidade de TC	“*Tem que ter um olhar mais aprofundado sobre a inovação*”; “*não há a cultura de empreendedorismo*”	6	16	13	35	12,0
Produtivismo acadêmico	Refere-se à hipervalorização da produção científica com baixo foco na implementação dos seus resultados para a sociedade	“*Uma avaliação do pesquisador baseada só na quantidade de artigos que ele publica, olha, isso realmente vai cair. A publicação virou um negócio, né? Publicação científica é um negócio altamente rentável, por sinal*”	0	2	3	5	1,7
Total			65	142	84	291	100,0

CT&I: ciência, tecnologia e informação; DI: características dos resultados de pesquisa; DII: cenário interno; DIII: cenário externo; ICT: instituto de ciência e tecnologia.

**Tabela 2:** Categorias dos fatores facilitadores à translação do conhecimento por domínio.

Categorização	Definição	Exemplos de relatos	DI	DII	DIII	Total	%
Infraestrutura e apoio institucional	Refere-se à existência de estruturas organizacionais que oferecem serviços e equipamentos para suporte à realização da pesquisa científica no ICT e à aplicação do conhecimento gerado (NIT, escritório de projetos, biotério, plataformas tecnológicas, entre outros)	“*as plataformas e os serviços multiusuários, acho que isso é um fator facilitador que a gente tem e às vezes até um fator de integração*”	2	23	4	29	43,9
Sistemas informatizados	Refere-se à oferta de sistemas operacionais informatizados que oferecem suporte técnico gerencial ao ICT	“*o Sistema de Gerenciamento de Projeto (SGP), os serviços internos disponibilizados na intranet, o Sistema de Apoio ao Planejamento Estratégico (SAGE) são exemplos de facilitadores*…”	0	2	0	2	3,0
Financiamento	Refere-se ao apoio financeiro disponibilizado por programas de incentivo ao ICT	“*Apoio de recursos financeiros obtidos junto ao NIH, MCTI (RenorBio), Ministério da Saúde, CNPq e Fapesb*”	5	5	0	10	15,2
Cooperação e parceria	Refere-se a colaborações formais e informais em apoio à pesquisa, desenvolvimento tecnológico e inovação	“*Colaboração interinstituições (Sesab, LACEN, UFBA, ONG Vontade de Viver), colaboração internacional (Instituto Pateur)”; “O primeiro passo que diria como facilitador é a nossa disposição de trabalhar junto com eles, trazer essa cooperação*”	3	3	2	8	12,1
Modelo de gestão participativa	Refere-se a requisitos de governança institucional, principalmente no tocante à forma como as lideranças são definidas, as decisões são tomadas e os fluxos de comunicação e informação são direcionados	“*…um fator facilitador de meu trabalho aqui é minha chefia então eu tenho uma chefia que além de ele acolher minhas ideias e de opinar e de sempre me dar bons conselhos e bons direcionamentos. Ele não é egoísta então ele deixa que realmente nós sejamos protagonistas, então isso pra mim é um fator de facilitação enorm*e”	0	7	0	7	10,6
Gestão das relações institucionais e governamentais	Refere-se às ações de interlocuções institucionais com entes públicos e privados, que objetivam facilitar a implementação dos resultados das evidências científicas produzidas pelo ICT	“*…reuniões de apresentação de prestação de contas realizadas pelo DECIT têm demonstrado interesse na identificação e análise dos resultados das pesquisas financiadas pelo órgão*”	1	1	5	7	10,6
Capacitação	Refere-se às ações de desenvolvimento profissional de diversas naturezas (cursos, congressos, treinamento em serviço e visitas técnicas).	“*Os programas de formação de recursos humanos do ICT são muito importantes para o desenvolvimento das pesquisas realizadas*”	1	1	0	2	3,0
Credibilidade da instituição	Refere-se ao reconhecimento da competência técnico-científica do ICT	“*Bom eu acho que facilita a marca do ICT né porque o nome tem um peso muito grande e aí você tem um peso, então eu acho que o nome do ICT é uma marca. Credibilidad*e”	0	0	1	1	1,5
Total			12	42	12	66	100,0

CNPq: Conselho Nacional de Desenvolvimento Científico e Tecnológico; DECIT: Departamento de Ciência e Tecnologia; DI: características dos resultados de pesquisa; DII: cenário interno; DIII: cenário externo; Fapesb: Fundação de Amparo à Pesquisa do Estado da Bahia; ICT: instituto de ciência e tecnologia; LACEN: Laboratório Central de Saúde Pública Noel Nutels; MCTI: Ministério da Ciência, Tecnologia e Inovação; NIH: Instituto Nacional de Saúde (Estados Unidos); NIT: núcleo de inovação tecnológica; ONG: organizações não governamentais; Sesab: Secretaria de Saúde do Estado da Bahia; UFBA: Universidade Federal da Bahia.

**Quadro 1:** Proposta de estratégias e abordagens de translação do conhecimento.

ESTRATÉGIAS E ABORDAGENS	ORIENTAÇÃO
Implantação de ferramenta de suporte automatizada	Desenvolver solução automatizada considerando funcionalidades que viabilizem a superação de barreiras e o fortalecimento dos fatores facilitadores identificados no contexto.
Definição de critérios para adequação de espaços	Definir critérios técnico-científicos para promover a otimização dos espaços físicos dedicados à realização das pesquisas e instalações dos equipamentos, adaptando-os às suas especificidades.
Disponibilização de equipamentos multiusuários e serviços técnico-científicos de apoio a pesquisa, desenvolvimento e inovação	Incentivar, fortalecer e priorizar a disponibilização de equipamentos multiusuários e serviços técnico-científicos de apoio à pesquisa, desenvolvimento e inovação (PDI).
Criação de instância de suporte à translação do conhecimento	Formalizar uma área com profissionais capacitados a oferecer apoio à translação do conhecimento.
Promoção do engajamento de partes interessadas	Realizar reuniões e/ou visitas com partes interessadas nos resultados obtidos nas pesquisas.
Definição da questão de pesquisa	Envolver os usuários do conhecimento no processo de concepção dos estudos, buscando priorizar suas necessidades e soluções para os problemas de saúde.
Realização de abordagem integrativa na fase inicial e na fase final da pesquisa	Incluir tomadores de decisão em discussões acerca de um problema potencial de pesquisa e nas atividades que ocorrem simultaneamente durante todo o processo de pesquisa; por exemplo, refinamento da questão de pesquisa, sugestões sobre o método, diálogo deliberativo e colaboração na interpretação dos dados coletados. Após a conclusão das pesquisas, realizar atividades que devem ir além da publicação de artigos, buscando implementar eventos, tais como café científico, *newsletter*s, *websites*, *blogs*, *policy briefs*, sessões de translação do conhecimento, vídeos, mídia social etc.
Inclusão da disciplina de translação do conhecimento nos programas de pós-graduação	Capacitar os pesquisadores no desenvolvimento de propostas de pesquisa com abordagem de translação do conhecimento; apoiar os membros do corpo docente da instituição nos esforços voltados à vinculação da pesquisa à ação; contribuir com a formação de novos pesquisadores criando vínculos entre a produção e aplicação do conhecimento.
Elaboração de materiais de divulgação de resultados de pesquisa	Desenvolver e formatar manuais, *kits* de ferramentas e outros materiais de apoio, em linguagem simples, de forma a tornar mais fácil para as partes interessadas entenderem e implementarem os resultados da pesquisa.
Elaboração de plano de implementação dos resultados de pesquisa	Desenvolver um plano de implementação formal que inclua metas, profissionais envolvidos e estratégias a serem utilizadas; criar e envolver um grupo formal de vários tipos de partes interessadas para fornecer informações e conselhos sobre os esforços de implementação e obter recomendações para melhorias.
Estímulo ao compartilhamento de recurso	Estimular colaborações no ambiente interno da instituição para compartilhamento e otimização de recursos e insumos de pesquisa.
Construção de redes de relacionamento	Construir redes de trabalhos no ambiente interno e externo da instituição para compartilhamento de informações, ideias e lições aprendidas referentes à implementação de inovações.
Criação e institucionalização de indicadores de implementação do conhecimento	Estabelecer indicadores para medir a capacidade do pesquisador e da instituição de implementação de evidências científicas.

Os relatos das entrevistas permitiram a identificação das categorias de barreiras apresentadas na Tabela 1, lideradas pelas categorias “financiamento em CT&I limitado” e “apoio técnico insuficiente para a translação do conhecimento”. No DI, observa-se que os entrevistados consideram nove categorias de barreiras, das quais a “falta de formação e desenvolvimento de competências para TC no ICT” teve maior frequência. O DII (ambiente interno) apresenta todas as 10 categorias de barreiras e o maior número de citações dos entrevistados, portanto, é o contexto com maior necessidade de intervenções. Ao analisar os desafios relatados pelos entrevistados referentes ao ambiente externo do ICT, percebe-se a força da barreira referente à “necessidade de gestão das relações institucionais e governamentais”, que se destaca com a maior quantidade de citações do DIII. Esses resultados são sustentados pela literatura que, de fato, pontua diversas dificuldades para a TC, desde formação de recursos humanos até políticas governamentais ^14^^,^^15^^,^^16^^,^^17^^,^^18^^,^^19^^,^^20^^,^^21^^,^^22^^,^^23^^,^^24^.

Adicionalmente, foram relatadas dificuldades decorrentes do desconhecimento das regulamentações e requisitos definidos e da demora no processo de análise da demanda, realizado pelos órgãos reguladores. A inexistência de uma instância com a missão de prestar apoio técnico especializado aos pesquisadores do ICT nas atividades necessárias à implementação dos resultados das pesquisas foi constatada como um grande desafio. O suporte à pesquisa é essencial, visto que libera o pesquisador da parte burocrática dos seus projetos, conforme salientado pelos entrevistados, e o tempo efetivamente gasto para realizar pesquisa, analisar os dados e publicar os resultados é bastante reduzido. De acordo com Siewert Junior & Parisotto ^31^, é necessário que os pesquisadores tenham acesso a essa estrutura, o que possibilita que haja um esforço maior na investigação. A dificuldade de acesso a evidências de pesquisa atualizadas, a escassa comunicação e colaboração existente entre as instituições de pesquisa e os que tomam decisões, e a distância entre as necessidades de conhecimento (dos pacientes, dos profissionais de saúde, dos governos e das instituições) e o trabalho dos pesquisadores ilustram os desafios que precisam ser ultrapassados, conforme percepção dos participantes deste estudo e coerente com os achados da literatura ^14^.

A categoria de barreira intitulada, neste estudo, como “produtivismo acadêmico” é caracterizada pelo autor Waters ^32^ como uma excessiva valorização da quantidade de produção científica-acadêmica. Como o autor afirma, existe uma ligação entre a demanda pelo aumento da produtividade e o esvaziamento de qualquer significado que não seja aumentar o número de publicações. No cenário brasileiro, o produtivismo acadêmico é alimentado por um processo de competição envolvendo universidades, docentes e pesquisadores, e pelo modelo de avaliação da ciência e do pesquisador, adotado pelo CNPq e outras agências de fomento, que têm no *Currículo Lattes* seu principal instrumento indicador de produtividade. O equilíbrio entre a produção e a recepção do conhecimento gerado foi perdido. Nesse sentido, considerando o foco da TC, é necessário que a interseção entre produção, aceitação e aplicação do novo conhecimento se torne cada vez mais integrada.

A Tabela 2 apresenta oito categorias de fatores que, de acordo com os pesquisadores entrevistados, facilitam a implementação dos resultados das pesquisas. Entre essas oito, a categoria relacionada à infraestrutura do instituto é considerada um fator facilitador principalmente pela existência do escritório de projetos, responsável pela gestão dos projetos de pesquisa, pelo núcleo de inovação tecnológica e por disponibilizar plataformas e serviços tecnológicos multiusuários. De acordo com as recomendações de Nilsen ^23^, esses facilitadores estão sendo considerados nas estratégias propostas para que sejam intensificados, a exemplo da criação de uma solução automatizada para suporte à aplicação dos resultados de pesquisa e à implantação de nova instância na infraestrutura, contendo as competências de apoio à pesquisa, o que fomenta as ações de capacitação defendidas por Andrade & Pereira ^22^.

Fundamentando-se em modelos teóricos de TC, este estudo buscou construir uma proposta - aplicável ao contexto de uma instituição pública de pesquisa no Brasil - que, combinando estratégias de governança e aplicação do conhecimento, resulta num modelo prático para favorecer a inovação em saúde. Os caminhos propostos nesta pesquisa pretendem aumentar a capacidade institucional de identificação e alinhamento às reais necessidades do sistema de saúde, facilitando a obtenção de resultados práticos decorrentes dos investimentos voltados à geração de conhecimento científico para solução de problemas de saúde da população. Com base nos dados coletados e analisados, foram desenvolvidas propostas de estratégias e abordagens para tratar os determinantes contextuais. O Quadro 1 apresenta essas sugestões com detalhamento de orientações para cada uma.

As estratégias propostas buscam contribuir para a superação das dificuldades e fortalecimento dos fatores facilitadores, e encontram fundamentação em Powell et al. ^33^, Lavis et al. ^12^ e Grimshaw et al. ^34^. Elas envolvem tanto a instituição e seus macroprocessos, como a atividade direta do pesquisador. Muitas das estratégias sugeridas já foram apontadas por outros estudos ^18^^,^^33^, como a “criação de redes de relacionamento” internas e externas e a “promoção do engajamento de partes interessadas” nos resultados de pesquisa. Algumas delas também vão ao encontro das tendências de inserção de tecnologias nos processos, como a “implantação de ferramenta de suporte automatizada”, adotada no modelo *integrated* de Lavis et al. ^12^, que permite a integração de esforços, por meio da adoção de uma plataforma de TC, e reforçada por recomendações feitas no estudo de Schmidt et al. ^35^. Destaca-se, também, a sugestão de estratégias que envolvem a formação e o desenvolvimento de competências para a TC, conforme já sinalizado em estudo realizado por Andrade & Pereira ^22^.

Ainda que essas estratégias tenham sido propostas por uma amostra restrita de pesquisadores de um ICT, suas recomendações, vindas de profissionais experientes, favorecem a identificação de informações referentes ao problema e ao contexto em que ele se encontra, ampliando a conscientização do que pode ser feito para uma resolução mais efetiva. Essas sugestões possibilitam implicações práticas para o desenvolvimento dos artefatos e implementação de inovações. Mesmo que outros estudos sejam necessários para o desenvolvimento e aprimoramento dos artefatos, inclusive com amostras ampliadas, os resultados aqui levantados são estruturantes e fundamentais para as próximas etapas da DSR, até que seja possível alcançar a meta final: fomentar a transformação do conhecimento científico gerado nas pesquisas em ações e produtos que contribuam para a melhoria da saúde populacional.

## Conclusão

A identificação das barreiras que, na visão dos pesquisadores, impedem a implementação do conhecimento resultante das pesquisas viabilizou a proposição de estratégias para diminuir a lacuna entre a criação do conhecimento e a sua aplicação prática. Os fatores facilitadores também foram importantes para o aprimoramento dessas estratégias. Os resultados contribuíram para ampliar a capacidade do ICT de transformar os conhecimentos gerados em práticas inovadoras que beneficiem a saúde da população brasileira

A disponibilização de uma instância organizacional dotada de visão estratégica e voltada ao apoio técnico especializado em inovação é uma estratégia indispensável para o aumento da contribuição dos institutos de pesquisa para a saúde. Essa instância, que deverá dispor de integrantes com as competências requeridas no processo de implementação de resultados de pesquisa, permitirá a liberação dos pesquisadores de funções burocráticas e políticas, possibilitando que esses profissionais dediquem mais tempo ao processo de criação de conhecimento.

Sendo assim, recomenda-se a implantação de um curso de formação em TC voltado ao público interno e externo do ICT, seja ele produtor ou consumidor de conhecimento científico, com o objetivo de formar profissionais para atuarem nos processos inerentes à conversão dos conhecimentos produzidos em inovação. No sentido de desenvolver a cultura da TC, sugere-se, ainda, a inclusão de um componente curricular nos programas de pós-graduação oferecidos pelo ICT, fortalecendo a familiaridade dos discentes e docentes com o tema já no processo de formação científica.

A criação de indicadores que demonstrem a capacidade de aplicação das evidências adquiridas nas investigações pode fortalecer o potencial de inovação dos pesquisadores, universidades e instituições de pesquisa, servindo como passo inicial para que seja revisto o modelo de avaliação da ciência, atualmente baseado fundamentalmente em dados bibliométricos, que incentivam o produtivismo acadêmico. Este estudo de caso também demonstra que as características dos resultados de pesquisa e o contexto interno do ICT afetam a adoção do processo de TC independentemente das restrições de recursos financeiros, em vista da diversidade de desafios apresentados.

Apesar de contar com uma amostra expressiva de 60% da população convidada para participar da entrevista, o fato de o estudo ter sido realizado em um único contexto pode ser considerado como uma limitação. Ampliar o número de contextos estudados e, consequentemente, a amostra é recomendado. Sendo assim, pesquisas futuras poderiam expandir os critérios de inclusão da amostra de pesquisadores, não restringindo apenas a bolsista de produtividade ou líderes de laboratório. A categorização realizada aqui pode ser usada com amostra ampliada, a fim de verificar se esses resultados se confirmam na percepção de diferentes pesquisadores e de outros ICT ou se novas estratégias podem ser adicionadas para o fomento da TC. Sugere-se, por fim, que sejam realizados estudos com o objetivo de conhecer o encaminhamento dado pelas agências de fomento aos conhecimentos produzidos nas pesquisas que financiam.

Por fim, grupos focais confirmatórios são bem recomendados na abordagem DSR e podem ser aplicados para análise da utilidade e replicabilidade dos artefatos (estratégias) em outras instituições. Além disso, como os artefatos aqui propostos não foram ainda implementados, destaca-se a continuidade deste estudo, avançando no desenvolvimento e aplicação das estratégias, seguindo, desse modo, com as demais etapas da DSR.
